# Preparation of Crystal Violet Lactone Complex and Its Effect on Discoloration of Metal Surface Coating

**DOI:** 10.3390/polym14204443

**Published:** 2022-10-20

**Authors:** Wenbo Li, Xiaoxing Yan, Wenting Zhao

**Affiliations:** 1 Co-Innovation Center of Efficient Processing and Utilization of Forest Resources, Nanjing Forestry University, Nanjing 210037, China; 2 College of Furnishings and Industrial Design, Nanjing Forestry University, Nanjing 210037, China

**Keywords:** thermochromic compound, preparation technology, coating process

## Abstract

In this paper, a thermochromic complex was prepared from crystal violet lactone (CVL), bisphenol A (BPA) and tetradecanol. The color-changing temperature of the color-changing compound was found to be 25 °C by orthogonal experiment. Microcapsules coated with a thermochromic compound were added into alkyd resin at different mass concentrations. With the increase in temperature and mass fraction of microcapsules in the coating, the color difference of the coating showed an upward trend. The highest variation in the coating’s color difference occurs when there were 10% microcapsules. When the mass fraction of microcapsules was 15.0~25.0%, there was little change to the gloss of the coating. With the increase in the mass fraction of the coating microcapsules, the hardness of the coating gradually increased. The hardness was at its best when the microcapsule concentration was 25%. When the microcapsule concentration was 20%, the impact resistance of the coating was at its best. The coating had good cold-liquid resistance to acetic acid, ethanol, and NaCl solutions, and there was basically no mark on the coating surface before and after the cold-liquid-resistance test. The addition of microcapsules did not change the chemical composition of the coating, and it improved the performance of the coating. When the microcapsule concentration was 10%, the overall performance of the coating was at its best, which laid the technical foundation for thermochromic coating on the metal surface.

## 1. Introduction

As a new type of functional coating, thermotropic reversible color-changing coating is widely used in industry, medical treatment, life, and other fields. Coating it on metal can form a protective, decorative, and functional coating [1,2]. Thermochromic materials are composed of chromogenic agents, cryptogenic agents, and solvents. The different proportions of these three components directly affect the final color-change effect and color development time [3,4]. The developer determines the type of color, the cryptogenic agents determine the depth of the color change, and the solvent determines the temperature of the color change. Because the temperature is controllable, the color selection is diverse [5,6]. Kingchok et al. [7] promoted local interactions and produced reversible thermal discoloration by combining zinc-aluminum layered double hydroxide (ZnAl-LDH) into p-Phenylenediamine (PDA) components, which can be used in intelligent coatings and colorimetric sensors. Muller et al. [8] applied thermochromic coating to a solar panel collector. The simulation results show that the balcony coated with a thermochromic coating can have a better heat-collection effect. Because the traditional type of color-changing coatings is mostly solvent-type color-changing coatings, the organic solvents are volatile, causing air pollution [9,10]. At the same time, color-changing materials are greatly affected by environmental factors, and their aging resistance and acid-based resistance are poor [11,12].

Pigment, as an important component of coating, is important to the performance of furniture made of metal and wood [13,14]. If the pigment is encapsulated, the wall material protects the pigment core material, and extends the service life [15]. The choice of microcapsule wall material has a great influence on the performance of the microcapsules [16,17]. The nature of wall material determines its flow and dispersion in the coating. Generally, the selection of wall materials can not react with core materials, and the wall materials should have better covering, sealing, and stability [18]. Urea formaldehyde resin is a good wall material for microcapsules because of its low price, wide source, and strong adhesion [19,20]. Tozum et al. [21] used a thermochromic complex prepared by CVL, BPA, and 1-tetradecanol, successfully prepared the microcapsules by lotion polymerization, and applied them to textiles. The results showed that the addition of microcapsules made the textile products have a sufficient heat resistance and cooling effect in the process of use. Zhu et al. [22] prepared thermochromic microcapsules with thermochromic compounds as core materials. Microcapsules were added to wood and wood coatings, and the thermochromic properties of the microcapsules were discussed. The results showed that the microcapsules can achieve thermochromic properties when combined with wood and paint. These studies show that the addition of thermochromic microcapsules can give the coating more excellent properties, but the different microcapsule contents have a great impact on the properties of the coating, which has not been widely reported.

In this paper, the mass ratios of CVL (aphakic agent): BPA (chromogenic agent) and CVL (aphakic agent): tetradecanol (solvent), as well as temperature and stirring time, were selected as influencing factors. An L_9_ (3^4^) orthogonal test with four factors and three levels was designed, and the discoloration temperature was taken as the experimental result to conduct experimental analysis, prepare a thermochromic complex, and determine the best preparation process for the discoloration complex. Then, the thermochromic complex was used as the core material to prepare thermochromic microcapsules. Different mass fractions of thermochromic microcapsules were added to the coating to coat the metal substrate. The optical, mechanical, cold-liquid resistance, and aging resistance of the coating were studied. The purpose for this was that the coating with microcapsules can improve the optical and mechanical properties of the coating, while having a good color-changing effect.

## 2. Materials and Methods

### 2.1. Test Materials

The experimental materials are shown in Table 1. Aluminum alloy plates with a smooth surface (50 mm × 50 mm × 0.5 mm) were bought from Jiangsu Daling metal materials Co., Ltd., Suzhou, China.

### 2.2. Preparation of Thermochromic Complex of CVL

According to the relevant literature [23], the mass ratio of CVL: BPA and the mass ratio of CVL: tetradecanol, as well as the temperature, and mixing time, were selected as the influencing factors, and the L_9_ (3^4^) orthogonal test with four factors and three levels was designed. The influencing factors and levels of the orthogonal test are shown in Table 2, and the arrangement of the orthogonal test is shown in Table 3.

Taking sample 1 # in Table 3 as an example, 200.0 g of tetradecanol was heated to 50 °C in a water bath to a molten state. A total of 10.0 g BPA and 5.0 g CVL were then added. A magnetic rotor was put into the water bath to stir. After uniformly stirring, the water bath was slowly heated to 70 °C. After stirring at 400 rpm for a certain time, the solution became clear and transparent. When it cooled to room temperature and turned into a dark-blue solid, it was a successfully prepared color-changing compound.

### 2.3. Preparation of Crystalline Violet Lactone Thermochromic Microcapsules

**Figure 1:**
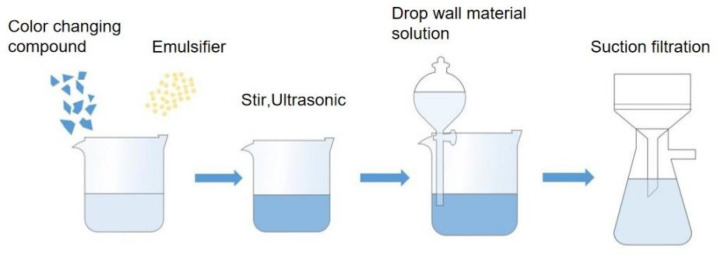
Microcapsule preparation process flowchart.

The process flowchart of microcapsule preparation is shown in Figure 1. A total of 8.00 g urea and 16.84 g formaldehyde were weighed in a beaker. A magnetic rotor was put into the beaker. The beaker was put in a thermostat water bath and stirred at a normal atmospheric temperature until the materials completely dissolved. Then, triethanolamine was dropped into the mixture to adjust the pH of the solution to about 8.5. Then, the temperature of the water bath was raised to 70 °C and stirred at 300 rpm for 1 h. Then, 288 g distilled water was added. A total of 5.48 g of Arabic gum powder emulsifier and 104.25 g of distilled water were weighed, and 8.23 g of the complex prepared by sample 6 # was added as the core material. Then, the temperature of the water bath was adjusted to 50 °C. It was stirred at a constant speed until the core material dissolved. Then, the water bath was adjusted to 65 °C and 1600 rpm, and the reaction continued for 20 min. The solution was emulsified for 5 min by an ultrasonic emulsifying machine. After ultrasonic treatment, the solution was placed at room temperature for a period of time, and then it was put into a 35 °C water bath to slowly stir. During the stirring process, the prepared wall material was dropped into the core material. After the dropwise addition, the stirring speed of the water bath was adjusted to 500 rpm, and 1.16 g of silica and 1.16 g of sodium chloride were added to prevent the prepared microcapsules from sticking together. By adding 8% citric acid monohydrate solution, the pH was adjusted to about 2.5. After reacting for 1 h, the temperature of the water bath was raised to 68 °C, and the stirred speed was adjusted to 250 rpm for 30 min to obtain microcapsule emulsion. After cooling, the emulsion was filtered, and the powder after drying was obtained as color-changing microcapsules [24,25].

### 2.4. Coating Preparation

First, the microcapsules and coatings were weighed and mixed according to Table 4. After stirring evenly, the mixture was painted on the surface of the metal substrate with an SZQ-type tetrahedral-coating preparation machine with an amount of 15~20 g/m^2^, according to QB/T 4461-2013 [26]. The samples were dried at room temperature for 1 h and then dried in an oven at 30 °C for 1 day. The thickness of the dried coating was about 60 μm. If the coating thickness is too thick, the coating is easy to crack, and if the coating thickness is too thin, the substrate cannot be protected.

### 2.5. Testing and Characterization

#### 2.5.1. Discoloration Temperature Test of CVL Thermochromic Complex

The solid color-changing compound was ground into small particles at room temperature. A total of 1.0 g of color-changing compound particles was weighed and put into a test tube. The water bath pot was heated, and the temperature was slowly raised from 20 °C to 2 °C each time. The solid color-changing compound lasted for 10 min at 25 °C to uniformly heat the discolored compound. When the compound began to change color, the temperature at this time was recorded [27].

#### 2.5.2. Chemical Composition Test of the Thermochromic Complex of CVL

A Vertex 80 V Fourier-transform infrared spectrometer (FTIR) was used to analyze the chemical composition of the microcapsules. A small amount of color-changing compound was put into a mortar mill, and a proper amount of potassium bromide (KBr) was added. After being fully ground under the irradiation of an ultraviolet lamp, it was poured into a grinding tool. It was put into an oil press for proper pressurization to turn it into a transparent sheet. As such, the sample preparation was completed, and subsequent tests could be carried out.

#### 2.5.3. Coating Testing and Characterization

According to GB/T 11186.3-1989 [28], the color difference of the coating was tested by an HP-2136 color-difference tester. The test parameters were 45° annular lighting, CIE 10° standard observer, and a CIE D65 light source. *L* is the brightness, *a* is the range from red to green, and *b* is the range from blue to yellow. The larger the *a* is, the redder it is, and the smaller the *a* is, the greener it is. The larger the *b* is, the yellower it is, and the smaller the *b* is, the bluer it is. The portable colorimeter was turned on, and the colorimeter was firstly calibrated. Then, the test hole with one part of the sample was aligned, the test key was pressed, and the values of *L*_1_, *a*_1_ and *b*_1_ were wrote down. Then, the test hole of the colorimeter with another part of the sample was aligned, the test key was pressed, and the values of *L*_2_, *a*_2_, and *b*_2_. were written down. The color difference (Δ*E*) was calculated using Formula (1) [29].

(1) $$\Delta E=\sqrt{{\left(L_{1}-L_{2}\right)}^{2}+{\left(a_{1}-a_{2}\right)}^{2}+{\left(b_{1}-b_{2}\right)}^{2}}$$

By using a heating plate, the sample was heated to test the thermochromic temperature of the coating. The gloss of the coating was measured by an HG268 glossmeter with GB/T 4893.6-2013 [30]. The hardness of the coating was tested by a portable coating hardness tester with GB/T 6739-2006 [31]. According to GB/T 4893.4-2013 [32], the adhesion of the coating was tested by a QFH-HG600 coating scribing instrument. According to GB/T 1732-1993 [33], the impact resistance of the coating was tested by the QCJ coating impact tester. Acetic acid, ethanol, coffee, and 15 wt.% NaCl solution were chosen as the cold-liquid-resistance testing agents of the coating to evaluate the coating’s cold-liquid resistance in accordance with GB/T4893.1-2005 [34]. Because these four liquids are acid, food, salt water and solvent, respectively, they are common liquids for representative wood-product-surface coatings. The coating’s center was chosen as the test area for its resistance to cold liquids. With tweezers, the filter paper from different testing agents was removed after being soaked for 5 s. It was placed on the coating surface, then a glass cover was covered on the testing sample surface for 24 h. After removing the glass cover and the filter paper, the remaining liquid was wiped off. Ultraviolet (UV) photooxidation aging resistance testing, according to GB/T 1865-2009 [35], and the artificially accelerated aging test (UV photooxidation) were performed in a UV weather-resistance test chamber (Nanjing Environmental Testing Equipment Co., Ltd., Nanjing, China). The irradiance of the xenon light was 50 W/m^2^. The film based on the metal substrate was placed in the UV test chamber. Every 24 h, until the coating had no discoloration performance, the chromaticity value of the coated surface was checked.

## 3. Results and Discussion

### 3.1. Discoloration Temperature and Composition Analysis of Discoloration Compound

**Figure 2:**
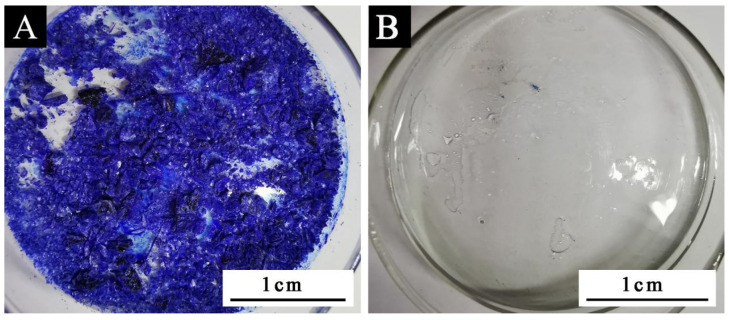
Schematic diagram of sample 6 #: (**A**) before discoloration and (**B**) after discoloration.

It can be seen from Table 5 that the minimum discoloration temperature of sample 6 # was 25 °C (Figure 2). According to the mean value and variance analysis in Table 6, it can be seen that the most influential factor on discoloration performance was CVL: BPA. When the mass ratio of CVL: BPA: tetradecanol was 1:3:60, and the reaction time was 1.5 h in a water bath at 90 °C, the color-changing compound had its best performance.

**Figure 3:**
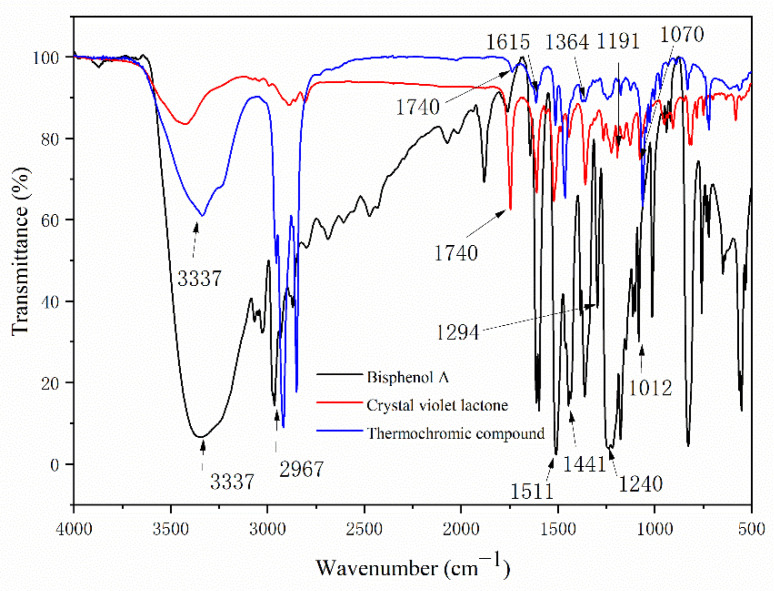
FTIR spectra of BPA, CVL, and the thermochromic compound.

It can be seen from Table 7 and Figure 3 that 3337 cm^−1^, 2967 cm^−1^, 1511 cm^−1^, 1441 cm^−1^, 1240 cm^−1^, 1294 cm^−1^, and 1012 cm^−1^ were, respectively, the telescopic peak of -OH, the telescopic peak of C-CH_3_, the telescopic peak of benzene ring C=C, the absorption peak of C-OH, and the telescopic peak of C-O, which belong to the characteristic peak of BPA [36,37]. The telescopic peak of the carbonyl group C=O of the lactone ring appeared at 1740 cm^−1^, and the symmetric telescopic peak of the ester group C-O-C appears at 1191 cm^−1^ and 1071 cm^−1^, which proves that the lactone ring was in a closed-loop state [38,39]. The -OH absorption peak at 3337 cm^−1^ of BPA in the infrared spectrum of the color-changing complex became weak, and most of them were characteristic peaks of crystalline violet lactone and BPA, which indicates that crystalline violet lactone only partially reacts with BPA to form a conjugated chromogenic system. In Figure 2, the stretching vibration absorption peak of the lactone ring structure C=O appeared at 1740 cm^−1^, the ester carbonyl C=O absorption peak appeared at 1615 cm^−1^, and the -OH absorption peak of carboxyl group appeared at 3337 cm^−1^, indicating that the lactone ring was broken, the conjugate system was destroyed, the structure was changed, and the color changed [40,41,42].

### 3.2. Influence of Color-Changing Microcapsules on the Color Difference of Coatings

**Figure 4:**
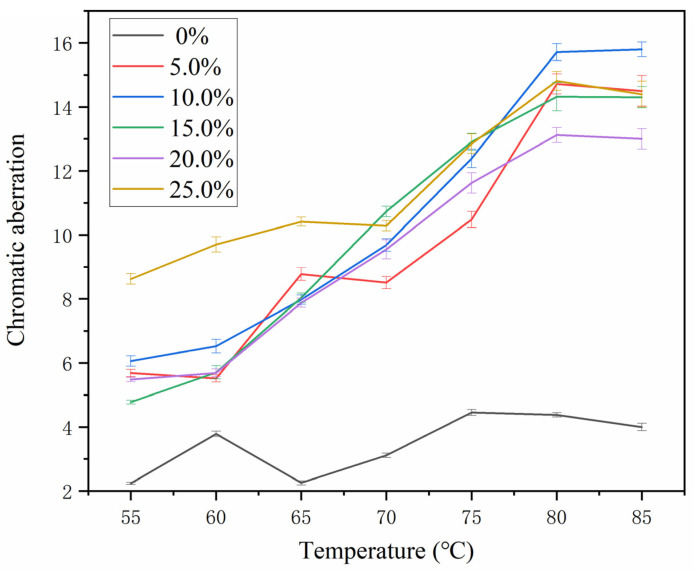
Effect of microcapsule content on the thermochromic difference of coating.

**Figure 5:**
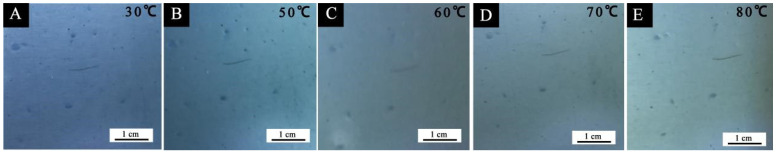
Discoloration of coating on a metal substrate with 10.0% thermochromic microcapsules: (**A**–**E**) color change during heating.

The chromatic parameters (*L*, *a*, *b*) and color differences (∆*E*) of the thermochromic microcapsules with different mass fractions on the metal surface are shown in Table 8 and Figure 4. The larger the ∆*E* value, the better the color-change effect of the coating. The figure shows that, with the addition of zero microcapsules, the coating’s color difference changes by about five, with little variation. With the same number of microcapsules added, the color difference of the coating increased as the temperature increased, and the coating’s color variation was greatest at 80 °C in temperature. With the increase in the mass fraction of the microcapsules, the color difference of the coating showed an upward trend. When 10.0% microcapsules were used, the color difference of the coating was 15.80 at 85 °C. Figure 5 shows the color-change effect of the coating of 10.0% thermochromic microcapsules from dark blue to light blue. When the number of microcapsules is lower than 10.0%, the microcapsules cannot be evenly distributed in the coating and cannot be completely coated on the surface of the substrate, so the color difference of the coating was smaller than that of 10.0%. When the number of microcapsules was large, although the substrate had a discoloration effect, the color difference did not increase significantly. This is because the transfer of temperature in the coating gradually decreased, and the temperature of the internal microcapsules in the coating was lower than the external temperature, so the discoloration effect of the coating was also poor when the number of microcapsules added was too large.

### 3.3. Effect of Microcapsule Mass Fraction on Coating Gloss

**Figure 6:**
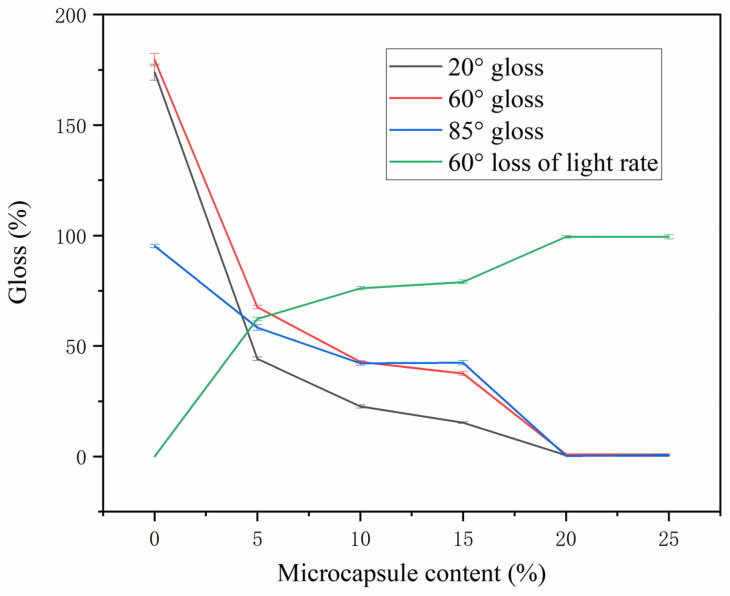
A gloss of coating with different microcapsule content.

The gloss of the test piece at 20°, 60° and 85° was measured by the gloss machine, and the gloss of the coating with microcapsules and the loss of light of the coating without microcapsules were calculated at an incidence angle of 60° [43]. The extinction ratio was calculated according to Formula (2). The influence of the mass fraction of different microcapsules on the coating gloss is shown in Table 9 and Figure 6. The gloss of the coating gradually decreased with the increase in the number of microcapsules added. When the number of microcapsules was 0–10.0%, the gloss of the coating changed greatly. When the mass fraction of microcapsules was 15.0–25.0%, the gloss of the coating changed gently. When the number of microcapsules was 5–15%, there was little change in the loss of light of the coating. When the number of microcapsules was 20–25%, the loss of light of the coating changed greatly. This is because the gloss and gloss loss of the coating are related to the flatness of the coating. When the number of microcapsules was too high, the flatness of the coating decreased, which reduced the light-reflection ability of the coating, thereby reducing the gloss and improving the gloss loss of the coating [44,45].

(2) $$G_{L}=\frac{\left(G_{0}-G_{1}\right)}{G_{0}}\times 100\%$$

where *G*_0_ is the coating without microcapsules, *G*_1_ is the coating put into the microcapsule, and *G_L_* is the extinction rate.

### 3.4. Effect of Mass Fraction of Microcapsules on Mechanical Properties of Coatings

Table 10 shows the effect of the addition of different microcapsules on the mechanical properties of the coating. The hardness of the coating increased with the increase in the mass fraction of microcapsules. When the mass fraction of microcapsules was 0–10.0%, the hardness of the coating was 2H. With the increase in the mass fraction of microcapsules, the hardness of the coating increased to 5H [46]. The coating adhesion grade of microcapsules with a mass fraction of 0–20.0% was considered grade 1. The reason for this is that the prepared microcapsules are small particles with a small particle size and good dispersion in the coating, which has little effect on the adhesion of the coating. When the mass fraction of the microcapsules in the coating was 5–15%, the impact resistance of the coating was at its best, which was 20 ± 1 kg∙cm. This is because the capsule wall of the microcapsule can effectively protect the capsule core to a certain extent. The coating with microcapsules was coated on the substrate, and the microcapsules can protect the coating, thereby improving the impact resistance of the coating [47,48].

### 3.5. Effect of Mass Fraction of Microcapsules on Cold-Liquid Resistance of Coatings

Table 11, Table 12 and Table 13 show the color difference, gloss, and cold-liquid-resistance grade of the coating before and after the cold-liquid resistance of the coating with different mass fractions of the microcapsules on metal substrates. The coating without microcapsules had a smooth surface, so the color difference of the coating was almost unchanged after the cold-liquid-resistance test. Acetic acid, NaCl solution, and ethanol had no obvious effect on the color change of the coating with the microcapsules. Due to the dark color of coffee, after the cold-liquid-resistance test, the traces on the coating surface were obvious. With the increase in the mass fraction of microcapsules, the traces gradually deepened and the color difference gradually increased. When the mass fraction of microcapsules in the coating was 25.0%, the color difference of the coating was at its largest. After the cold-liquid-resistance test, there was little change to the gloss of the coating. The coating had good cold-liquid resistance for the other three solutions, except for coffee, and the cold-liquid-resistance grade was grade 1. The coating had poor cold-liquid resistance to coffee. With the increase in the mass fraction of microcapsules, the cold-liquid-resistance grade increased, and the coating surface traces were obvious. When the mass fraction of microcapsules was 15–25%, the cold-liquid resistance of the coating was at its worst, and the cold-liquid-resistance grade decreased to grades 2 and 3. This is because, with the increase of microcapsules, the coating surface becomes uneven and coffee flows into it. After a period of time, the coating surface leaves stains [49,50].

### 3.6. Effect of Mass Fraction of Microcapsules on Aging Resistance of Coatings

Table 14 shows the chromaticity values and color differences before and after the aging of the coating, with and without the thermochromic microcapsules. The coating with the microcapsules was blue before aging and white after aging, and the color difference was 7.00. After aging, the coating had no discoloration properties. This is because, under the continuous irradiation of ultraviolet light, the internal structure of the chromotropic compound and crystal violet lactone was destroyed, so the coating no longer had a color-changing performance. After the UV-aging process, the color difference of the coating without microcapsules was 6.15. This is because the components in the alkyd resin film-forming materials are sensitive to ultraviolet rays and are prone to degradation and cross-linking reactions under ultraviolet irradiation, resulting in the irreversible yellowing of the coating [51,52].

### 3.7. Structure and Composition Analysis of Color-Changing Coating

**Figure 7:**
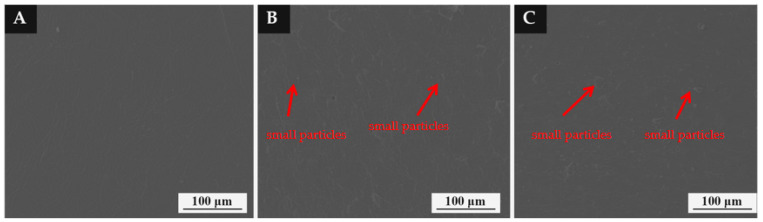
Morphology of coating with different content of microcapsules: (**A**) 0%, (**B**) 10.0%, (**C**) 15.0%.

Figure 7 shows the SEM morphology of the coating with different mass fractions of thermochromic microcapsules on different substrates. The surface of the coating without microcapsules was smooth. When the mass fraction of microcapsules in the coating increased to 10.0% and 15.0%, the micro morphology of the coating was relatively smooth. The reason for this is that microcapsules are small particles. When the number of microcapsules was low, it had little effect on the smoothness of the coating.

**Figure 8:**
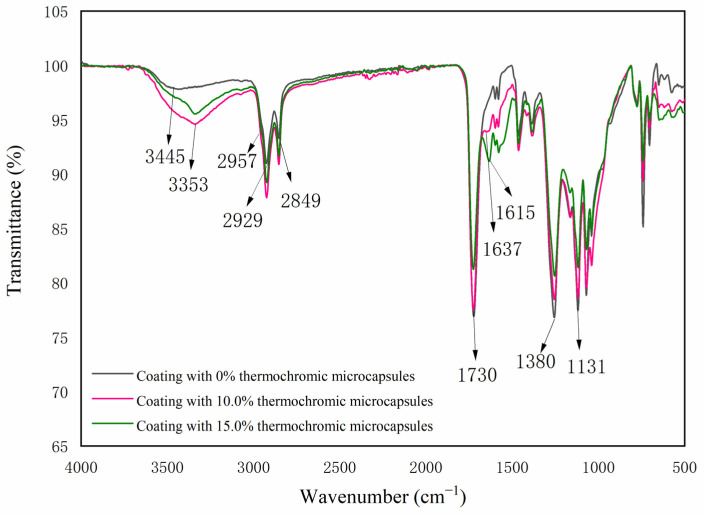
FTIR spectra of coatings with different microcapsule contents.

Figure 8 shows the infrared spectra of the thermochromic coating with different contents of microcapsules. The coating without the microcapsules had a telescopic peak of the hydroxyl group at 3445 cm^−1^, a characteristic peak of C=O in ester bond at 1730 cm^−1^, and a characteristic peak of C-H in the methyl and methylene groups at 2929 cm^−1^ and 2849 cm^−1^, which are characteristic peaks of alkyd resin coating [53]. The telescopic peak of the N-H bond and the O-H bond was 3353 cm^−1^, 2957 cm^−1^ was the asymmetric stretching vibration of -CH_2_-, 1637 cm^−1^ was the carbonyl telescopic peak of secondary acyl, and 1131 cm^−1^ was the characteristic absorption peak of CH_3_O. The appearance of these peaks indicated that urea formaldehyde resin had been successfully prepared. At 1615 cm^−1^, the non-lactone ring structure’s ester carbonyl C=O absorption peak can be seen, while 1380 cm^−1^ is where the carboxylate’s symmetric stretching absorption peak can be found. The results indicate that no chemical reaction occurs between the microcapsules and alkyd resin paint.

## 4. Conclusions

Through the orthogonal test of four factors and three levels, the mass ratio of CVL:BPA:tetradecanol was 1:3:60. When the reaction time was 1.5 h in the water bath at 90 °C, the discoloration temperature of the discoloration complex was 25 °C. When 10.0% microcapsules were added, the color difference of the coating was the most obvious. When the mass fraction of the microcapsules was 25.0%, the hardness of the coating was at its best, the adhesion level of the coating increases, and the impact resistance first increased and then decreased. When the mass fraction of the microcapsules in the coating was 20.0%, the impact resistance of the coating was at its best. The cold-liquid resistance of the coating for coffee was poor. When the mass fraction of microcapsules was 10.0%, the performance of the coating was at its best. At this time, the color-change temperature of the coating was 80 °C, the color difference was 15.71 ± 0.78, the gloss was 42.8 ± 2.1%, the hardness was 2 H, the adhesion was grade 1, and the impact resistance was 20 ± 1 kg·cm. With the increase in the mass fraction of the microcapsules, the cold-liquid-resistance grade of the coating increased, and the surface traces of the coating were more obvious. The liquid-resistance grade was grade 1 for acetic acid, ethanol, and NaCl solution. The results provide a technical reference for the application of thermochromic microcapsules on metal substrates.

## Figures and Tables

**Table 1:** List of experimental materials.

Experimental Materials	Molecular Mass (g/mol)	CAS	Manufacturer
CVL	415.52	1552-42-7	Wuhan Huaxiang Biotechnology Co., Ltd., Wuhan, China
BPA	228.28	80-05-7	Shanghai sea area chemical Co., Ltd., Shanghai, China
Tetradecanol	214.38	112-72-1	Guangzhou Jiangshun Chemical Technology Co., Ltd., Guangzhou, China
37.0% formaldehyde	30.03	50-00-0	Shandong xinjiucheng Chemical Technology Co., Ltd., Jinan, China
Triethanolamine	149.19	102-71-6	Shandong Chengkai New Material Co., Ltd., Linyi, China
Urea	60.06	57-13-6	Guangzhou Suixin Chemical Co., Ltd., Guangzhou, China
Citric acid monohydrate	502.51	99026-99-0	Jinan Xiaoshi Chemical Co., Ltd., Jinan, China
Absolute ethanol	46.06	64-17-5	Guangzhou Chengyi Nuoyi Instrument Co., Ltd., Guangzhou, China
Acetic acid	60.05	64-19-7	Jinan Xiaoshi Chemical Co., Ltd., Jinan, China
Hydrochloric acid	36.46	7647-01-0	Jinan Xiaoshi Chemical Co., Ltd., Jinan, China
Gum acacia	-	9000-01-5	Nanjing Jinyou Biotechnology Co., Ltd., Nanjing, China
Alkyd resin paint	-	-	Zhejiang Daqiao paint Co., Ltd., Huzhou, China

**Table 2:** Influencing factors and levels.

Level	CVL:BPA	CVL:Tetradecanol	Temperature (°C)	Mixing Time (h)
1	1:2	1:40	70	1.0
2	1:3	1:50	80	1.5
3	1:4	1:60	90	2.0

**Table 3:** Orthogonal experiment table.

Sample (#)	CVL:BPA	CVL:Tetradecanol	Temperature (°C)	Mixing Time (h)
1	1:2	1:40	70	1.0
2	1:2	1:50	80	1.5
3	1:2	1:60	90	2.0
4	1:3	1:40	80	2.0
5	1:3	1:50	90	1.0
6	1:3	1:60	70	1.5
7	1:4	1:40	90	1.5
8	1:4	1:50	70	2.0
9	1:4	1:60	80	1.0

**Table 4:** Experimental material list.

Mass Fraction of Microcapsules (%)	Microcapsule Weight (g)	Coating Weight (g)
0	0	2.0
5.0	0.1	1.9
10.0	0.2	1.8
15.0	0.3	1.7
20.0	0.4	1.6
25.0	0.5	1.5

**Table 5:** Visual analysis table.

Sample (#)	Leucophore: Developer	Leucophore: Solvent	Temperature (°C)	Time (h)	Discoloration Temperature (°C)
1	1:2	1:40	70	1:2	33
2	1:2	1:50	80	1:2	34
3	1:2	1:60	90	1:2	30
4	1:3	1:40	80	1:3	29
5	1:3	1:50	90	1:3	28
6	1:3	1:60	70	1:3	25
7	1:4	1:40	90	1:4	29
8	1:4	1:50	70	1:4	30
9	1:4	1:60	80	1:4	31
Mean value 1	32.333	30.333	29.333	30.667	
Mean value 2	27.333	30.667	31.333	29.333	
Mean value 3	30.000	28.667	29.000	29.667	
Range	5.000	2.000	2.333	1.334	

**Table 6:** Analysis of variance table.

Factor	Sum of Squares of Deviations	Freedom	F Ratio	F Critical Value	Significance
Leucophore: Developer	37.556	2	13.000	9.000	*
Leucophore: Solvent	6.889	2	2.385	9.000	
Temperature (°C)	9.556	2	3.308	9.000	
Time (h)	2.889	2	1.000	9.000	
Error	2.89	2			

Note: * in Table 6 means significant.

**Table 7:** Characteristic peaks of the infrared spectrum.

Peak Value (cm^−1^)	Characteristic Peak	Substance	Cause of Formation
3337	-OH	BPA	telescopic peak
2967	C-CH_3_	BPA	telescopic peak
1511 and 1441	Benzene ring C=C skeleton	BPA	telescopic peak
1240	C-OH	BPA	Absorption peak
294 and 1012	C-O	BPA	telescopic peak
1740	Lactone cyclocarbonyl C=O	CVL	telescopic peak
1191 and 1071	Ester group C-O-C	CVL	Symmetric telescopic peak
1615	Ester carbonyl of non-lactone ring structure C=O	Color-changing compound	Absorption peak
3337	-OH in the carboxyl group	Color-changing compound	Absorption peak

**Table 8:** Chromaticity value and color difference of coatings with different microcapsule contents as temperature increases.

Microcapsule Content (%)	Color Parameter	55 °C	60 °C	65 °C	70 °C	75 °C	80 °C	85 °C
0	*L*	35.30 ± 1.76	34.60 ± 1.73	34.70 ± 1.73	36.20 ± 1.81	36.20 ± 1.81	34.00 ± 1.70	35.10 ± 1.75
	*a*	22.30 ± 1.11	24.80 ± 1.24	22.50 ± 1.12	20.20 ± 1.01	24.70 ± 1.23	25.00 ± 1.25	24.60 ± 1.23
	*b*	15.00 ± 0.75	14.40 ± 0.72	15.80 ± 0.79	15.40 ± 0.77	14.20 ± 0.71	15.60 ± 0.78	15.80 ± 0.79
	∆*E*	2.23 ± 0.11	3.78 ± 0.18	2.25 ± 0.11	3.11 ± 0.15	4.45 ± 0.22	4.38 ± 0.21	4.00 ± 0.2
5.0	*L*	64.40 ± 3.22	65.30 ± 3.26	67.10 ± 3.35	66.10 ± 3.30	66.00 ± 3.30	71.50 ± 3.57	70.50 ± 3.52
	*a*	−0.70 ± 0.03	0.00 ± 0.00	−0.65 ± 0.03	0.05 ± 0.00	0.70 ± 0.03	−1.08 ± 0.05	0.95 ± 0.04
	*b*	8.90 ± 0.44	8.25 ± 0.41	10.90 ± 0.54	11.40 ± 0.57	13.70 ± 0.68	15.00 ± 0.75	15.70 ± 0.78
	∆*E*	5.68 ± 0.28	5.51 ± 0.27	8.77 ± 0.43	8.51 ± 0.42	10.48 ± 0.52	14.71 ± 0.73	14.50 ± 0.72
10.0	*L*	66.20 ± 3.31	64.80 ± 3.24	65.20 ± 3.26	65.00 ± 3.25	66.40 ± 3.32	68.00 ± 3.40	65.10 ± 3.25
	*a*	−2.30 ± 0.11	−1.40 ± 0.07	−1.40 ± 0.07	−1.30 ± 0.06	−0.60 ± 0.03	−1.20 ± 0.06	−1.10 ± 0.05
	*b*	8.60 ± 0.43	12.10 ± 0.60	13.90 ± 0.69	16.00 ± 0.80	18.90 ± 0.94	21.90 ± 1.09	22.70 ± 1.13
	∆*E*	6.06 ± 0.30	6.52 ± 0.32	7.97 ± 0.39	9.68 ± 0.48	12.39 ± 0.61	15.71 ± 0.78	15.80 ± 0.79
15.0	*L*	57.70 ± 2.88	60.30 ± 3.01	58.30 ± 2.91	57.30 ± 2.86	57.30 ± 2.86	59.70 ± 2.98	60.30 ± 3.01
	*a*	−3.90 ± 0.19	−3.80 ± 0.19	−3.80 ± 0.19	−3.60 ± 0.18	−3.40 ± 0.17	−2.40 ± 0.12	−2.10 ± 0.10
	*b*	6.80 ± 0.34	9.30 ± 0.46	11.90 ± 0.59	14.50 ± 0.72	16.60 ± 0.83	18.10 ± 0.90	17.80 ± 0.89
	∆*E*	4.77 ± 0.23	5.70 ± 0.28	8.03 ± 0.40	10.73 ± 0.53	12.90 ± 0.64	14.32 ± 0.71	14.20 ± 0.71
20.0	*L*	58.60 ± 2.93	58.70 ± 2.93	58.30 ± 2.91	57.60 ± 2.88	59.90 ± 2.99	61.20 ± 3.06	58.70 ± 2.93
	*a*	−3.10 ± 0.15	−2.60 ± 0.13	−3.20 ± 0.16	−2.30 ± 0.11	−1.90 ± 0.09	−1.70 ± 0.08	−1.60 ± 0.08
	*b*	4.50 ± 0.22	4.50 ± 0.22	7.30 ± 0.36	9.10 ± 0.45	10.50 ± 0.52	11.60 ± 0.58	12.30 ± 0.61
	∆*E*	5.48 ± 0.27	5.68 ± 0.28	7.87 ± 0.39	9.54 ± 0.47	11.62 ± 0.58	13.12 ± 0.65	13.00 ± 0.65
25.0	*L*	64.00 ± 3.20	63.00 ± 3.15	64.30 ± 3.21	63.10 ± 3.15	65.50 ± 3.27	66.10 ± 3.30	67.30 ± 3.36
	*a*	−1.90 ± 0.09	−2.70 ± 0.13	−1.80 ± 0.09	−2.20 ± 0.11	−1.40 ± 0.07	−1.60 ± 0.08	−1.70 ± 0.08
	*b*	2.80 ± 0.14	4.60 ± 0.23	4.70 ± 0.23	5.20 ± 0.26	6.80 ± 0.34	8.70 ± 0.43	7.60 ± 0.38
	∆*E*	8.62 ± 0.43	9.70 ± 0.48	10.41 ± 0.52	10.28 ± 0.51	12.85 ± 0.64	14.81 ± 0.74	14.40 ± 0.72

**Table 9:** The gloss of coating with different microcapsules content.

Mass Fraction of Microcapsules (%)	20° (%)	60° (%)	85° (%)	Extinction Rate (%)
0	173.9 ± 8.6	179.5 ± 8.9	95.2 ± 4.7	-
5.0	44.2 ± 2.2	67.6 ± 3.3	58.3 ± 2.9	62.3 ± 3.1
10.0	22.6 ± 1.1	42.8 ± 2.1	42.1 ± 2.1	76.1 ± 3.8
15.0	15.3 ± 0.7	37.6 ± 1.8	42.4 ± 2.1	79.0 ± 3.9
20.0	0.5 ± 0.0	0.9 ± 0.0	0.3 ± 0.0	99.4 ± 4.9
25.0	0.4 ± 0.0	0.9 ± 0.0	0.6 ± 0.0	99.4 ± 4.9

**Table 10:** Mechanical properties of coating with different microcapsules content.

Mass Fraction of Microcapsules (%)	Hardness	Adhesion (Level)	Impact Strength (kg·cm)
0	2H	0	18.0 ± 0
5.0	2H	1	20.0 ± 1
10.0	2H	1	20.0 ± 1
15.0	3H	1	20.0 ± 1
20.0	4H	1	18.0 ± 0
25.0	5H	2	17.0 ± 0

**Table 11:** The liquid-resistant color difference of coating with different microcapsules contents.

Mass Fraction of Microcapsules (%)	Cold-Liquid-Resistance Color Difference			
	Acetic Acid	Coffee	NaCl Solution	Ethanol
0	1.8 ± 0	4.9 ± 0.2	2.4 ± 0.1	3.6 ± 0.1
5.0	12.6 ± 0.6	2.7 ± 0.1	6.7 ± 0.3	18.5 ± 0.9
10.0	7.3 ± 0.3	4.4 ± 0.2	4.1 ± 0.2	2.1 ± 0.1
15.0	5.4 ± 0.2	5.5 ± 0.2	9.1 ± 0.4	8.5 ± 0.4
20.0	4.0 ± 0.2	10.5 ± 0.5	7.4 ± 0.3	1.8 ± 0
25.0	3.8 ± 0.1	13.2 ± 0.6	3.0 ± 0.1	1.9 ± 0

**Table 12:** Liquid gloss resistance of coating with different microcapsule contents.

Mass Fraction of Microcapsules (%)	Gloss before Cold-Liquid Resistance	Cold-Resistant Gloss (%)			
		Acetic Acid	Coffee	NaCl Solution	Ethanol
0	179.5 ± 8.8	172.5 ± 8.6	170.2 ± 8.5	170.5 ± 8.5	175.8 ± 8.7
5.0	67.6 ± 3.3	68.4 ± 3.4	62.6 ± 3.1	67.5 ± 3.3	63.9 ± 3.1
10.0	42.8 ± 2.1	49.3 ± 2.4	46.0 ± 2.3	47.0 ± 2.3	47.6 ± 2.3
15.0	37.6 ± 1.8	40.8 ± 2.0	36.2 ± 1.8	35.0 ± 1.7	37.9 ± 1.8
20.0	0.9 ± 0	1.2 ± 0	1.0 ± 0	0.8 ± 0	0.9 ± 0
25.0	0.9 ± 0	0.9 ± 0	1.0 ± 0	1.1 ± 0	1.0 ± 0

**Table 13:** Liquid-resistance grade of coating with different microcapsule content.

Mass Fraction of Microcapsules (%)	Cold-Liquid-Resistance Grade (Level)			
	Acetic Acid	Coffee	NaCl Solution	Ethanol
0	1	1	1	1
5.0	1	1	1	1
10.0	1	1	1	1
15.0	1	2	1	1
20.0	1	2	1	1
25.0	1	3	1	1

**Table 14:** Color-difference change after aging test of coating with 0 and 10% microcapsule addition.

Microcapsule Content (%)	State	*L*	*a*	*b*	∆*L*	∆*a*	∆*b*	Chromatic Aberration
0	Before aging	81.05 ± 4.07	−6.43 ± 0.32	29.15 ± 1.45	1.05 ± 0.05	−1.93 ± 0.09	5.75 ± 0.28	6.15 ± 0.30
	After aging	80.00 ± 4.00	−4.50 ± 0.22	23.40 ± 1.17				
10.0	Before aging	63.35 ± 3.16	2.95 ± 0.14	7.45 ± 0.37	2.25 ± 0.11	3.35 ± 0.16	−5.72 ± 0.28	7.00 ± 0.35
	After aging	61.10 ± 3.05	−0.40 ± 0.02	13.17 ± 0.65				

## Data Availability

The data presented in this study are available on request from the corresponding author.
